# Relationship between view of context, psychosocial malaise and problematic internet use: mediation analysis using partial least squares structural equation modelling

**DOI:** 10.1192/bjo.2022.517

**Published:** 2022-06-30

**Authors:** Lucrezia Ferrante, Claudia Venuleo, Giovanna Alessia Sternativo, Simone Rollo, Jun-Hwa Cheah, Sergio Salvatore, Enrico Ciavolino

**Affiliations:** Department of History, Society and Human Studies, University of Salento, Lecce, Italy; School of Business and Economics, Universiti Putra Malaysia, Seri Kembangan, Selangor, Malaysia; Department of Dynamic and Clinical Psychology, University of Roma ‘La Sapienza’, Rome, Italy; and Department of History, Society and Human Studies, University of Salento, Lecce, Italy

**Keywords:** Problematic internet use, adolescents, sense-making, psychosocial malaise, cultural context

## Abstract

**Background:**

According to more recent approaches on problematic internet use (PIU), using the internet can be seen as a way of compensating for psychosocial malaise. Taking semiotic cultural psychology theory as its theoretical framework, this study examines the role of affect-laden assumptions concerning the world, known as latent dimensions of sense (LDSs), in promoting (or not) adaptive responses, including internet use as a maladaptive strategy against problems and difficulties.

**Aims:**

To test a theoretical model in which PIU is predicted by LDSs through the mediation of high levels of psychosocial malaise.

**Method:**

We measured PIU (using the Generalized Problematic Internet Use Scale 2), LDSs (View of Context questionnaire), negative affect (Positive and Negative Affect Schedule), social anxiety (Interaction Anxiousness Scale) and loneliness (Italian Loneliness Scale) in 764 Italian adolescents (mean age 15.05 years, s.d. = 1.152 years). LDSs were detected using a multiple correspondence analysis; after confirmatory composite analysis, partial least squares structural equation modelling with higher-order components was performed to test the mediation model.

**Results:**

The results show a relationship between LDSs corresponding to an extreme negative evaluation of the sociocultural context, experienced as absolutely unreliable, and PIU through the mediation of psychosocial malaise (95% CI 0.101– 0.171; *P* = 0.000).

**Conclusions:**

Overall, the findings suggest that PIU might be a way of compensating for unpleasant states in a context perceived in an extremely negative and homogenising way, i.e. as totally lacking resources and trustworthy people.

The internet is an integral element of today's life, not just allowing people to interact each other, but especially offering potentially unlimited possibilities in commercial, entertainment, information as well as professional and academic fields. Its rapid and pervasive spread has raised questions among scholars about its impact on people's health; the condition of a clinically significant impairment in the personal, social and academic/work spheres is known as problematic internet use (PIU).^1^

Despite there being no consensus on conceptualisation of PIU,^2^ a well-recognised aspect is that it may serve as a way of coping with unpleasant moods and experiences.^3^

Prevalence estimates vary considerably in PIU studies, ranging from 1 to 36%, probably reflecting population differences, plurality of instruments and different criteria for PIU behaviours.^4^ According to the European Parliamentary Research Service,^5^ the majority of individuals with problems related to internet use are adolescents. In Italy, the context of the current study, there is a substantial lack of studies assessing the prevalence of PIU in adolescent samples.

One of the most popular interpretations of adolescents’ vulnerability to problematic behaviours sees teenagers as having characteristics connected to their developmental age that make them vulnerable to substance and behavioural addictions. Although there is no doubt that adolescents share some characteristics (e.g. the level of development of the nervous system or increased levels of sensation-seeking and greater difficulties in emotion regulation) that differentiate them from other groups,^6^ the insistence on common aspects is also critical, because it tends to obscure differences related to psychosocial factors that may play a role in exposing them to the risk of PIU.^7^ A compensating hypothesis describing the link between social variables and extensive media use was first introduced by Davis & Kraus;^8^ more recently, Kardefelt-Winther^9^ proposed a ‘compensatory internet use model’, which explains PIU development and maintenance as the result of an unhealthy, persistent, rigid use of the internet to deal with negative affective experiences, such as social anxiety and depression.^10^ Several studies have considered the role of intrapsychic factors in adolescent PIU, such as depression and anxiety, personality traits and low self-esteem.^11^ Other studies addressed the role of factors related to the interpersonal sphere, such as family and school environment.^12^^,^^13^

Less attention has been paid to the role of the sociocultural environment in increasing psychosocial malaise and in constructing the meaning of internet use. In this paper, we argue that this role needs to be considered for at least two reasons. First, the sociocultural environment itself may act as a source of malaise when it constrains people's lives and lacks resources and opportunities (e.g. formative and professional chances; recreational settings such as cinema, theatres, cultural associations) essential for young people's growth, including supportive social networks. Internet use may be a way to satisfy sociability and identity needs. Second, the cultural environment offers the semiotic resources and cues underlying ways of perceiving and experiencing and therefore dealing with the social world.^14^ For instance, it is reasonable that person A, who sees the social environment as devoid of development opportunities and inhabited by selfish people, and person B, who expresses trust in people from their community and sees the context as full of resources, express different levels of social malaise, as well as a different way of using the internet, for instance as a maladaptive strategy against feelings/situations perceived as unsustainable.

Framed within semiotic cultural psychology theory,^14^ the present study postulates and emphasises the need to consider the deep interconnection between the individual and society in understanding maladaptive patterns of behaviour like PIU. A semiotic, cultural and psychosocial perspective allows us to reflect on the role of cultural context, understood as a net of interconnected meanings, grounding the way of perceiving and experiencing a social environment, and enabling individuals to orient themselves in their material and social world; in other words, such an approach leads to investigating PIU not as the result of an intrapsychic structure, rather as the precipitate of a specific modality of relationship between the individual and the meanings active within the semiotic context in which they are inscribed.^14^

Not neglecting the role of both the individual and relational dimensions, the study aims to contribute to the existing debate on adolescent PIU by considering sociocultural features in terms of adolescents’ views of their context. Indeed, available resources and opportunities, needs and motivation, social values and norms may act as constraints on the multiple ways adolescents can think and act, including the willingness to engage in problematic behaviours to compensate for their malaise. Recognising that the individual level dynamically interacts with the sociocultural level can offer a contribution to the way PIU – as well as problematic behaviours in general – can not only be understood, but also addressed: if PIU is not just a matter of individual health, multi-level interventions will be able to consider the individual and systemic dimensions in integrated and synergic ways.^15^

## Semiotic cultural psychology theory

Semiotic cultural psychology theory argues for the role of the sense-making process in the way people view and deal with the social world.^14^ According to this perspective, people give meaning to their life events in terms of symbolic resources (beliefs, knowledge, values) grounded on implicit, generalised world-views (symbolic universes, in the terms of the theory), made up by affect-laden basic latent dimensions of sense (LDSs).^14^ The LDSs have a bipolar structure, for example Salvatore and colleagues^16^ mapped three LDSs: pleasant versus unpleasant, passivity versus engagement and demand for systemic resources versus demand for community bond.

LDSs are not a property of the individual, do not emerge in a social vacuum, but are the by-product of a dynamic process (sense-making), where individuals, embedded in a specific system (e.g. family, school, workplace) and culture recursively interact with each other. Broader contextual dimensions (e.g. policies in the health and economic field, media communication, scientists’ and politicians’ discourses) set boundaries on the attribution of meaning to the events, difficulties, challenges and conditions of their lives. Previous studies have shown the relationship between a negative way of perceiving the social environment – namely as an anomic place with no future and no one to rely on – and problematic behaviours such as harmful drinking, smoking, gambling, as well PIU itself.^17,18^

Semiotic cultural psychology theory highlights two aspects of LDSs related to their capacity to promote adaptive responses.

The first is their variable degree of salience.^19^ Interpretations of reality characterised by a high salience correspond to a rigid, polarised way of thinking, so that objects and situations are homogenised (typically, organised by the friend/enemy, pleasure/displeasure opposition) without considering their particular nature. Such homogenising interpretations of reality are associated with a reduced capacity to regulate thoughts and behaviours on the basis of social constraints and requirements. Conversely, interpretations of reality characterised by a low salience are associated with a way of thinking able to differentiate objects of experience. Flexible thinking is also able to produce a plurality of meanings and allows personal and/or community resources to be used more effectively. Barrett and colleagues^20^ expressed a similar concept, suggesting that individuals with poorly differentiated emotional experiences are less able to regulate emotions and respond adaptively to events. Previous studies support the hypothesis of a significant relationship between the degree of affective salience and adaptation, showing how drug addicts, problem gamblers and internet users reported global, homogenising and generalising embodied affect-laden interpretations of reality, at the cost of more fine-grained and differentiated analytical thought. The scientific literature recognises that problematic behaviour is often linked to emotional processes, suggesting that the lower the ability to modulate the valence and intensity of one's emotional experience, the higher the risk of problem behaviour,^21^ including PIU.^22^

The second is the degree to which the beliefs, feelings and actions underpinned by LDSs are consistent with interpersonal and social tasks, rules and goals. Similarly, the notion of ‘cultural consensus’^23^ highlights that levels of psychosocial distress as well as the risk of harmful behaviours may be the result of low proximity with widely shared cultural models.

**Fig. 1:**
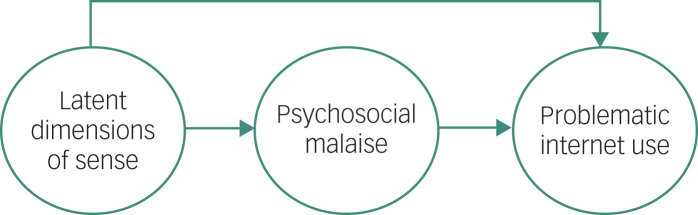
The theoretical model: problematic internet use is predicted by latent dimensions of sense through the mediation of high levels of psychosocial malaise.

Based on semiotic cultural psychology theory and the view of PIU as a compensating mechanism, the present study aims to explore a theoretical model (Fig. 1) in which PIU is predicted by the LDSs grounding adolescents’ view of the world – through the mediation of high levels of psychosocial malaise. Our hypothesis is that homogenised affect-laden interpretations of the context and a low identification with social tasks, rules and goals may fuel negative affect, which in turn increases the risk of PIU. Indeed, within a negative and distrustful view of the relational and social context, internet use can be seen as the only way to cope with problems in life.

Psychological malaise is detected considering adolescents’ levels of loneliness, social anxiety and negative emotions: indeed, internet use can be a mood-regulation strategy when experiencing an unpleasant state and a way to compensate for the sense of isolation.^10^

## Method

### Participants

Public high schools situated in southern Italy (the Salento area) were contacted by a letter describing the purpose of the study. Four schools agreed to participate, three situated in the city of Lecce, one in a smaller town of the province. In the schools participating, grade 9 classes, attended by adolescents from 13 to 15 years of age, and grade 11 classes, attended by adolescents from 16 to 19 years of age, were selected for the study so as to include the age group with the highest rates of PIU in Europe.^24^ In each school, four classes were randomly selected. Parental consent was required for students under 18: between 1 and 2% of the parents did not give consent.

In total, 764 students (mean age 15.05 years, s.d. = 1.152 years) were involved in the study. The sociodemographic characteristics of the sample, disaggregated by gender, are shown in Table 1. Significant differences related to gender were found for the school year attended.

**Table 1:** Sociodemographic characteristics of the sample

	Male, %	Female, %	Total, *n*	χ^2^
Age, years				
13–15	54.1	45.90	427	0.356
16–19	51.93	48.07	337	
Class				
Year 9	53.40	46.60	397	0.022
Year 11	52.86	47.14	367	

### Instruments

#### Problematic internet use (PIU)

The Generalized Problematic Internet Use Scale 2 (GPIUS-2)^3^ was used for self-assessment of PIU; the scale consists of 15 items rated on an eight-point Likert scale (from ‘definitely disagree’ to ‘definitely agree’). Its Italian version^25^ includes four factors: preference for online social interaction (POSI), mood regulation (MORE), deficient self-regulation (DSRE) and negative outcome (NOUT).

#### Latent dimensions of sense (LDSs)

The View of Context (VOC)^26^ questionnaire was used to map the LDSs through which people interpret their social context. Respondents are asked to report their opinions about the social environment, for instance by evaluating the place where they live, or the degree of reliability of services (e.g. health services and schools), as well as to state the moral/social values in which they believe, for instance studying or respecting each other. The questionnaire is composed of 45 items associated with a four-point Likert scale (‘not at all’, ‘not much’, ‘quite a lot’, ‘a lot’ or ‘very unreliable’, ‘rather unreliable’, ‘quite reliable’, ‘very reliable’) and exploring three main domains: ‘reliability of the context’, ‘agency’ and ‘success’.

#### Psychosocial malaise

We used three instruments to assess aspects of psychosocial malaise:

- the Interaction Anxiousness Scale (IAS),^27^ with its 15 items rated on a five-point Likert scale (from ‘not at all characteristic of me’ to ‘extremely characteristic of me’), was used to measure the levels of anxiety in social interaction; the IAS validated in Italy considers a higher-order latent variable, with four lower-order latent variables;^28^
- the Negative Affect subscale from the Italian version^29^ of the Positive and Negative Affect Schedule (PANAS)^30^ was employed to investigate the presence of negative affect; the respondent self-reports how much his or her experience is consistent with the adjective reported, ranging on a 5-point Likert scale (from ‘not at all’ to ‘very much’;
- the General Loneliness subscale from Italian Loneliness Scale (ILS),^31^ a self-report questionnaire for subjective loneliness. ILS General Loneliness consists of seven items rated on a four-point Likert scale (from ‘I often feel this way’ to ‘I never feel this way’).

### Procedure

Google Forms was used to administer, via computer, the set of instruments used for data collection. Two members of the research team introduced the study, highlighting that the voluntary nature of participation and the anonymity of responses were assured. Participants were also informed that the data would be analysed collectively and that only the research team would have access to them. Participants aged 18 and over provided their written informed consent to participate in the study. A parent or guardian of participants under the age of 18 provided written informed consent. Participants spent an average of 30 min responding. All procedures used in this research comply with the ethical standards of the relevant national and institutional committees on human experimentation and with the Helsinki Declaration of 1975, as revised in 2008. All procedures involving human participants/patients were approved by the Ethics Commission for Research in Psychology of the Department of History, Society and Human Studies of the University of Salento (25 March 2021; protocol no. 0056300).

### Data analysis

#### Detection of the LDSs

Variability in responses on the VOC was considered in order to obtain LDSs.^26^ For instance, a statement such as ‘I can only count on myself’ may convey the symbolic meaning of ‘agency’ if it is associated with statements such as ‘life is determined by actions’. However, the same expression ‘I can only count on myself’ may convey the meaning of ‘anomie’ if it is associated with statements such as ‘to succeed in life, it is important to have few scruples’.

Multiple correspondence analysis (MCA)^32^ can be used to map such variability. It consists of an explorative technique that enables LDSs to be defined by extracting a limited number of factorial dimensions to sum up the relations observed between nominal or ordinal data. To explore the possible patterns existing among the answers related to adolescents’ representations of their context, we carried out an MCA with the variables from the VOC questionnaire, namely ‘reliability of the context’, ‘agency’, and ‘success’. Each factorial dimension extracted describes the juxtaposition of two patterns of strongly associated response modes, corresponding to the opposition between two generalised meanings. This means that a factorial polarity collects the modalities of responses that are linked together as the effect of an LDS, independent of its specific content/semantic relationship. Therefore, a single answer (e.g. ‘the police are not reliable at all’) can occur in more than one dimension, but what makes it interpretable is the way such a statement is linked to the other answers – grouped on the same factor – in a pre-semantic, affect-laden way.

The respondent's position (coordinate) on the main factorial dimension is considered: a higher score indicates high proximity between the respondent's responses and the affect-laden interpretations underpinning that factorial dimension. This analysis was run on the software SPAD 8.2 for Windows (https://ia-data-analytics.com/data-mining-software/).

#### Mediation model for higher-order modelling with PLS-SEM

**Fig. 2:**
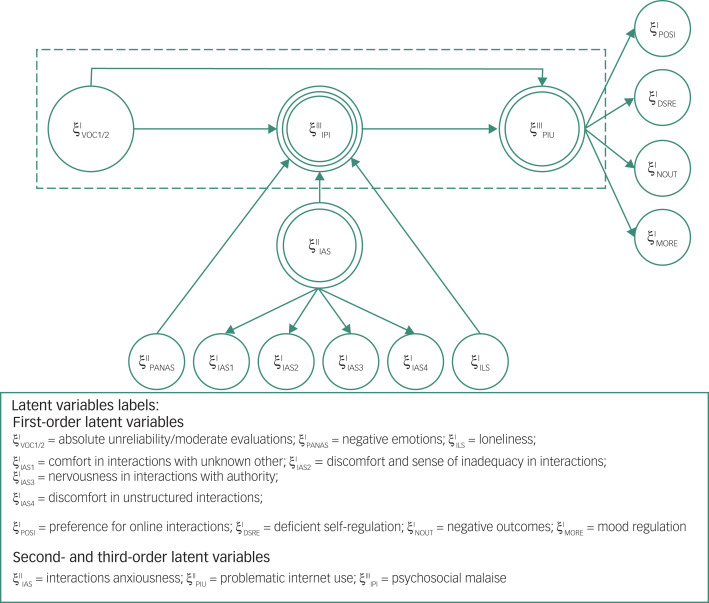
Mediation model for higher-order modelling. POSI, preference for online social interaction; DSRE, deficient self-regulation; VOC, View of Context questionnaire; IPI, psychosocial malaise; PIU, problematic internet use; NOUT, negative outcome; IAS, Interaction Anxiousness Scale; MORE, mood regulation; PANAS, Positive and Negative Affect Schedule; ILS, Italian Loneliness Scale.

To explore the mediation effects, it is necessary to have strong *a priori* theoretical support, which can be formalised by a direct or indirect effect between two variables.^33^ In the proposed mediation model (Fig. 2), LDSs predict PIU through the mediation of psychosocial malaise. Moreover, psychosocial malaise and PIU are defined with higher-order modelling, where the higher-order component represents a multidimensional concept that occurs at a certain level of abstraction, which is related to lower-order components representing their underlying and more concrete subdimension.^34^ The model is thus defined considering as the dependent variable ξ^III^PIU, expressed as a second-order latent variable, measured through the first-order latent variables POSI, DSRE, NOUT and MORE.

The measurement of the higher-order models can be reflective–reflective or formative–reflective when the higher-order component represents a more general construct simultaneously explaining all the underlying lower-order components, and reflective–formative or formative–formative when the lower-order components form the general higher-order component without sharing a unique cause.^33–35^

In our model, two types were adopted:

- Reflective–reflective, for the second-order latent variables named IAS and PIU. This choice finds a justification in the theoretical concepts behind social anxiousness (measured on the IAS)^28^ and problematic use of the internet (PIU).^25^ They are measures representing the effects of an underlying first-order latent variable. Therefore, since a reflective measure dictates that all indicators are caused by the same construct (psychosocial malaise and PIU), their indicators should be highly correlated with each other. Finally, individual items should be interchangeable, and any single item can be left out without changing the principal construct.^33^
- Reflective–formative, for the third-order latent variable named psychosocial malaise. The adoption of the reflective–formative model is justified by the theoretical concept behind the higher-order component psychosocial malaise, which is formed by the three lower-order components ILS, PANAS and IAS. Each dimension can vary independently of the others, i.e. the specific lower-order components (PANAS, IAS and ILS) do not have a unique and shared cause, but they form the general higher-order component.

To estimate the mediation model, structural equation modelling based on partial least squares (PLS-SEM)^36^ with higher-order components was implemented using Smart PLS 3.3.2 for Mac OSX (www.smartpls.com) and R 4.1.0 for Mac OSX (www.r-project.org). The measurement model evaluation was performed via confirmatory composite analysis (CCA).^37^ In addition, the embedded two-stage approach^38^ was used for specifying and estimating higher-order latent variables (psychosocial malaise and PIU) in PLS-SEM, which is appropriate for reflective–formative type higher-order components,^34^ in particular when a component, such as psychosocial malaise, plays a mediation role in the nomological network as defined in Fig. 2. The two stages of this approach are as follows.

The first stage coincides with the standard repeated indicators approach. This step is used for the assessment of the measurement model and to estimate the scores of the lower-order components used in the second stage as indicators of the higher-order components.

In the second stage the estimated scores in the previous step define the indicators (manifest variables) of the higher-order components. Specifically, the third-order psychosocial malaise component will be formatively measured using the estimated scores on the IAS, PANAS and ILS. The second-order component PIU is measured by the estimated scores for POSI, DSRE, NOUT and MORE. The parameter estimation in this step was performed by considering the consistent version of the PLS estimator (PLSc) in such a way as to reduce the bias of the formative measurement model. PLSc provides a correction for estimates of the path coefficients, construct correlations and indicator loadings.^39^

#### Assessment of the measurement model with CCA

To assess the higher-order measurement model, a two-stage process has to be followed.

In the first stage the lower-order reflective measurement model (IAS 1/2/3/4, ILS, PANAS, POSI, MORE, DSRE, NOUT) is assessed considering the internal consistency (composite reliability), convergent validity (average variance extracted, AVE), discriminant validity and indicator loading and their significance (bootstrap procedure).

In the second stage the higher-order measurement model (psychosocial malaise, IAS and PIU) is assessed as follows:

- the third-order formative construct (psychosocial malaise) is assessed by considering as items the lower-order components scores, interpreting the relationships between this and lower-order components as weights, and assessing convergent validity (AVE), collinearity between indicators (variance inflation factor, VIF) and significance and relevance of the outer weights (bootstrap procedure);
- the second-order IAS and PIU constructs are assessed through the internal consistency (composite reliability), convergent validity (AVE), discriminant validity and indicator loading and their significance (bootstrap procedure).

#### Assessment of structural model

PLS-SEM results are evaluated considering the coefficients of determination (*R*^2^) and the significance of the path coefficients, together with the *f^2^* effect sizes, predictive relevance (*Q*^2^) and *q^2^* effect sizes.

Assessment of PLS-SEM outcomes has been extended examining the mediating effects of psychosocial malaise on the relationship between the view of context (VOC1 and VOC2 extracted through MCA) and PIU.

## Results

### Detection of LDSs

After applying the formula of inertia adjustment,^40^ it was found that the first factorial dimension of the VOC (VOC1) accounts for 48.62% and the second (VOC2) for 16.41%, thus the two factors account for 65.03% of the total inertia.

#### VOC1: models of relationship with the social environment

This dimension (Table 2) contrasts two patterns of answers interpreted as two models of relationship with one's social environment: moderate reliability (−) versus absolute unreliability (+). On the moderate reliability polarity, moderate answers co-occur, i.e. items associated with intermediate points on a Likert scale (e.g. ‘somewhat agree’, ‘somewhat disagree’); such items concern the future, the possibility of acting to change the state of things, and the degree of trustworthiness of people and institutions. The absolute unreliability polarity collects items that adopt extreme scores on Likert scales (e.g. ‘strongly agree’, ‘not at all’) and have negative content (e.g. services and institutions are not available, it is not possible to change in the future, there is no faith in the recovery of the country). An anomic view emerges, where the only rule seems to be not having scruples and siding with the strongest.

**Table 2:** Response modes most significantly associated with the first factorial dimension (VOC1) of the View of Context questionnaire

Test value	Item	Response
**Moderate reliability**		
−10.77	The future will be	Somewhat better
−9.49	Life under control of powerful others	Somewhat disagree
−9.18	To succeed in life is a matter of luck	Somewhat disagree
−9.11	It is useless to bustle	Somewhat disagree
−9.04	Life is determined by actions	Somewhat agree
−9.01	To succeed in life, alliances are important	A little
−8.85	Companies are	Quite reliable
−8.45	To succeed in life, sharing is important	Somewhat
−8.44	It is possible to break rules for loved ones	Somewhat agree
−8.33	To succeed in life, understanding the world is important	Somewhat
−8.30	Life depends on accidental happenings	Somewhat disagree
−8.21	School is	Quite reliable
−7.67	Public offices are	Quite reliable
−7.65	Asking help of people in public roles is useless	Somewhat agree
−7.55	People are unable to change	Somewhat disagree
−7.47	I do not know who I could count on	Somewhat disagree
**Absolute unreliability**		
13.09	I do not know who I could count on	Strongly agree
12.92	Asking help of people in public roles is useless	Strongly agree
12.27	The Church is	Not at all reliable
11.87	It is possible to break rules for loved ones	Strongly agree
11.49	Public offices are	Not at all reliable
10.74	School is	Not at all reliable
10.26	To succeed in life, it is important having few scruples	Very
10.09	You can only live day by day	Strongly agree
10.03	People are unable to change	Strongly agree
9.82	It is impossible to make provisions for future	Strongly agree
9.76	To succeed in life, alliances are important	Very
9.53	The life of the people is getting worse	Strongly agree
9.48	To succeed in life, it is important following rules	Not at all
9.41	It is useless to bustle	Strongly agree
9.40	The police is	Not at all reliable
9.38	To succeed in life is a matter of luck	Strongly agree
9.35	In the next 5 years the place where I live will be	Much worse

#### VOC2: modes of evaluating the social environment

The opposite patterns of responses of the second factorial dimension (Table 3) were interpreted in terms of two ways of evaluating the social environment: idealising (−) versus moderate (+). Overall, the response modality is reflected in the tendency to generalise positive evaluations of objects and situations or to modulate judgements.

**Table 3:** Response modes most significantly associated with the second factorial dimension (VOC2) of the View of Context questionnaire

Test value	Item	Response
**Idealising**		
−12.81	School is	Very reliable
−12.34	To succeed in life, it is important acquiring knowledge	Very
−11.19	To succeed in life, it is important to follow rules	Very
−10.44	Healthcare services are	Very reliable
−9.76	To succeed in life, it is important sharing	Very
−9.33	The police is	Very reliable
−9.16	The Church is	Very reliable
−8.80	It is useless to bustle	Strongly disagree
−8.63	Public offices are	Very reliable
−8.32	Life is under control of powerful others	Strongly disagree
−8.10	The future will be	Much better
−8.01	It is hardly fair having children	Strongly disagree
−7.48	People are unable to change	Strongly disagree
−7.33	Public transport is	Very reliable
**Moderate**		
10.59	To succeed in life, it is important to acquire knowledge	Somewhat
7.95	The Church is	Not at all reliable
7.43	To succeed in life, it is important following rules	A little
6.95	The police is	Not very reliable
6.94	Public offices are	Not at all reliable
6.83	In the next 5 years the place where I live will be	Somewhat worse
6.71	Healthcare services are	Not very reliable
6.63	It is impossible to make provisions for future	Somewhat agree
6.37	Life depends on accidental happenings	Somewhat agree
6.26	To succeed in life, it is important sharing	A little
6.19	Public offices are	Not very reliable
6.11	The police is	Not at all reliable
6.03	School is	Not at all reliable
5.96	People are unable to change	Somewhat agree
5.95	Healthcare services are	Not at all reliable
5.84	Public transport is	Not very reliable
5.79	School is	Not very reliable
5.55	The future will be	Somewhat worse

On the idealising polarity, responses associated with extreme points on the Likert scales are aggregated (e.g. ‘very’, ‘much better’), with a set of homogeneously positive evaluations which depict the context as a pleasant place: there is faith that the future will be better, people are considered able to change, importance is given to rules and knowledge. On the moderate polarity, responses associated with the intermediate point on the Likert scales are aggregated (e.g. ‘not very’, ‘somewhat’). The content of the items is both positive and negative: on the one hand, some elements of the macro social environment are perceived as tending to be untrustworthy; on the other hand, traces of faith in people's possibility to improve their condition are expressed.

### Assessment of the measurement model with CCA

#### First stage

Table 4 reports the results of the lower-order reflective measurement model. All the standardised outer loadings of the latent variables are above the threshold values (0.40–0.708), with significant bootstrap intervals, which suggests a sufficient level of indicator reliability. Composite reliability for all the latent variables involved in this study exceeded the threshold of 0.70 and the AVE value above 0.50 suggests an adequate convergent validity for all the constructs except for IAS (AVE = 0.493), which can be very well approximated to 0.50, and PANAS (AVE = 0.396), the values of which in several studies^41,42^ have ranged from 0.17 to 0.43.

**Table 4:** Assessment of factor loadings, composite reliability (CR) and convergent validity (AVE)

Lower-order components	Items	Factor loadings	Bootstrap 95% CI	CR	AVE
Comfort in interactions with unknown other (IAS1)	IAS_2R	0.755	(0.659– 0.833)	0.743	0.493
	IAS_3R	0.746	(0.650–0.819)		
	IAS_15R	0.593	(0.447–0.712)		
Discomfort and sense of inadequacy in interactions (IAS2)	IAS_9	0.738	(0.691–0.776)	0.811	0.518
	IAS_11	0.739	(0.694–0.778)		
	IAS_12	0.709	(0.652–0.750)		
	IAS_13	0.694	(0.640–0.738)		
Nervousness in interactions with authority (IAS3)	IAS_4	0.771	(0.391–0.551)	0.757	0.515
	IAS_8	0.798	(0.750–0.832)		
	IAS_14	0.560	(0.465–0.641)		
Discomfort in unstructured interactions (IAS4)	IAS_1	0.713	(0.654–0.758)	0.752	0.503
	IAS_5	0.683	(0.608–0.743)		
	IAS_7	0.732	(0.666–0.774)		
Loneliness (ILS)	ILS_1	0.592	(0.530–0.644)	0.885	0.527
	ILS_2	0.750	(0.708–0.783)		
	ILS_3	0.791	(0.754–0.819)		
	ILS_4	0.710	(0.665–0.746)		
	ILS_5	0.658	(0.606–0.705)		
	ILS_6	0.793	(0.754–0.822)		
	ILS_7	0.762	(0.719–0.795)		
Preference for online interactions (POSI)	GPIUS_1	0.586	(0.502–0.654)	0.760	0.527
	GPIUS_3	0.808	(0.777–0.833)		
	GPIUS_13	0.745	(0.705–0.779)		
Mood regulation (MORE)	GPIUS_4	0.708	(0.632–0.765)	0.845	0.646
	GPIUS_5	0.853	(0.821–0.879)		
	GPIUS_8	0.843	(0.809–0.868)		
Deficient self-regulation (DSRE)	GPIUS_2	0.699	(0.644–0.738)	0.859	0.504
	GPIUS_6	0.682	(0.632–0.731)		
	GPIUS_9	0.740	(0.692–0.773)		
	GPIUS_10	0.642	(0.571–0.694)		
	GPIUS_11	0.760	(0.711–0.797)		
	GPIUS_12	0.730	(0.688–0.767)		
Negative outcome (NOUT)	GPIUS_7	0.869	(0.761–0.833)	0.832	0.624
	GPIUS_14	0.691	(0.844–0.889)		
	GPIUS_15	0.800	(0.623–0.745)		
Negative emotions (PANAS)	PANAS_1	0.636	(0.578–0.687)	0.864	0.396
	PANAS_2	0.702	(0.648–0.744)		
	PANAS_3	0.744	(0.700–0.775)		
	PANAS_4	0.733	(0.692–0.765)		
	PANAS_5	0.689	(0.630–0.733)		
	PANAS_6	0.712	(0.668–0.749)		
	PANAS_7	0.483	(0.393–0.554)		
	PANAS_8	0.400	(0.313–0.467)		
	PANAS_9	0.581	(0.517–0.634)		
	PANAS_10	0.507	(0.418–0.579)		

AVE, average variance extracted; IAS, Interaction Anxiousness Scale; ILS, Italian Loneliness Scale. GPIUS, Generalized Problematic Internet Use Scale, version 2; PANAS, Positive and Negative Affect Schedule.

Table 5 shows the results of the Fornell & Larcker^43^ criterion assessment: the square roots of the reflective lower-order construct AVE (shown in bold) are all higher than the correlations with the other latent variables. This means that all the constructs are able to measure unique concepts.

**Table 5:** Assessment of discriminant validity^a^

	DSRE	IAS1	IAS2	IAS3	IAS4	ILS	MORE	NOUT	PANAS	POSI
DSRE	**0.710**									
IAS1	0.080	**0.702**								
IAS2	0.261	0.278	**0.720**							
IAS3	0.146	0.054	0.465	**0.718**						
IAS4	0.231	0.209	0.592	0.311	**0.709**					
ILS	0.246	0.177	0.431	0.198	0.435	**0.726**				
MORE	0.612	0.081	0.134	0.103	0.147	0.170	**0.804**			
NOUT	0.769	0.079	0.197	0.106	0.167	0.174	0.645	**0.790**		
PANAS	0.318	0.078	0.398	0.384	0.410	0.392	0.205	0.229	**0.629**	
POSI	0.747	0.054	0.246	0.167	0.191	0.258	0.597	0.645	0.281	**0.719**

DSRE, deficient self-regulation; IAS, Interaction Anxiousness Scale; ILS, Italian Loneliness Scale; MORE, mood regulation; NOUT, negative outcome; PANAS, Positive and Negative Affect Schedule; POSI, preference for online social interaction.

a. Entries highlighted in bold show the square root of the convergent validity (average variance extracted, AVE). The other entries show the bivariate correlations between the constructs.

#### Second stage

The higher-order measurement model (IAS, PIU and psychosocial malaise) is assessed as follows.

For the second-order reflective IAS and PIU constructs, composite reliability values are respectively 0.791 and 0.923, exceeding the threshold of 0.70. The AVE values are respectively 0.504 and 0.749, above the threshold of 0.50, suggesting an adequate convergent validity for both second-order latent variables. In this case too, the bootstrap method was used with 1000 subsamples and we found that all relationships are significant (*P* < 0.05) for both IAS and PIU. Table 6 reveals that all weights of reflective indicators have significant *t*-values so that all the indicators can be maintained; additionally, confidence intervals and *t*-values for reflective indicators also support the significance of weights (i.e. 0 did not occur between the higher and lower values). The second-order discriminant validity of PIU and IAS is shown in Table 7.

**Table 6:** Second-order (IAS and PIU) estimated loadings and bootstrap results

	Original Sample	Sample mean	s.d.	*t*-statistic	*P*	95% CI
IAS → IAS1	0.400	0.401	0.055	7.307	0.000	(0.274–0.500)
IAS → IAS2	0.909	0.908	0.008	112.266	0.000	(0.891–0.923)
IAS → IAS3	0.649	0.651	0.032	20.199	0.000	(0.578–0.705)
IAS → IAS4	0.781	0.781	0.017	46.955	0.000	(0.742–0.809)
PIU → DSRE	0.933	0.934	0.005	178.434	0.000	(0.922–0.942)
PIU → MORE	0.800	0.800	0.018	44.124	0.000	(0.759–0.833)
PIU → NOUT	0.881	0.881	0.010	85.407	0.000	(0.859–0.900)
PIU → POSI	0.843	0.843	0.012	72.941	0.000	(0.817–0.863)

IAS, Interaction Anxiousness Scale; PIU, problematic internet use; DSRE, deficient self-regulation; MORE, mood regulation; NOUT, negative outcome; POSI, preference for online social interaction.

**Table 7:** Assessment of second-order discriminant validity^a^

	Interaction Anxiousness Scale	Problematic internet use
Interaction Anxiousness Scale	**0.520**	
Problematic internet use	0.263	**0.653**

a. Entries highlighted in bold show the square root of the convergent validity (AVE). The other entry shows the bivariate correlation between the constructs.

For the third-order formative construct (psychosocial malaise) assessment, considering the constructs IAS, PANAS and ILS as predictors of psychosocial malaise, the VIF values are less than the threshold of 5 (Table 8), therefore collinearity is not an issue between the constructs’ formative indicators.

**Table 8:** Variance inflation factors for the third-order formative constructs

Formative constructs	Variance inflation factor
Social anxiety (IAS)	1.473
Loneliness (ILS)	1.441
Negative emotions (PANAS)	1.367

IAS, Interaction Anxiousness Scale; ILS, Italian Loneliness Scale; PANAS, Positive and Negative Affect Schedule.

Through the bootstrapping procedure and using 1000 resamples, the indicators’ weights are well above the recommended value of 0.1. Table 9 reveals that all weights of formative indicators have significant *t*-values, which provides empirical support for retaining all the indicators; the confidence intervals as well as *t*-values for the formative indicators provided additional evidence regarding the significance of weights, as 0 did not occur between the higher and lower values of the confidence intervals.

**Table 9:** Third-order (psychosocial malaise) estimated loadings and bootstrap results

	Original sample	Sample mean	s.d.	*t*-statistic	*P*	95% CI
IAS → psychosocial malaise	0.402	0.402	0.015	26.186	0.000	(0.373–0.432)
ILS → psychosocial malaise	0.416	0.416	0.015	27.604	0.000	(0.389–0.448)
PANAS → psychosocial malaise	0.441	0.441	0.017	26.280	0.000	(0.409–0.475)

IAS, Interaction Anxiousness Scale; ILS, Italian Loneliness Scale; PANAS, Positive and Negative Affect Schedule.

Convergent validity cannot be performed without global or multiple items for redundancy analysis.

The ‘variance-explained’ (*R*^2^) value of the endogenous variable psychosocial malaise is 0.133, exceeding the threshold of 0.10. It shows a good proportion of variance in the dependent variable explained by the independent variable.

The predictive validity (*Q*^2^), assessed through the blindfolding method, was obtained through a cross-validated redundancy procedure. Table 10 shows that 0.230 was the *Q*^2^ value for psychosocial malaise, which represents high relevance for the endogenous construct (i.e. psychosocial malaise).

**Table 10:** Third-order (psychosocial malaise) estimated loadings and blindfolding results

	SSO	SSE	*Q*² (=1 − SSE/SSO)
IAS	9.932.000	9.784.329	0.015
ILS	5.348.000	4.993.133	0.066
Psychosocial malaise	22.920.000	17.639.913	**0.230**
PANAS	7.640.000	7.493.344	0.019

Bold denotes high relevance for the endogenous construct; SSE, sum of squares error; SSO, sum of squares of observations; IAS, Interaction Anxiousness Scale; ILS, Italian Loneliness Scale; MORE, mood regulation; NOUT, negative outcome; PANAS, Positive and Negative Affect Schedule.

### Measurement model evaluation in mediation analysis

In the literature there are three types of mediation: complementary (or partial mediation), competitive (or partial mediation) and indirect-only (or full) mediation.^34^

The evaluation of mediation analysis was conducted via the bootstrap procedure.

Table 11 reports the bootstrap results for the direct and indirect mediation effects of VOC1. The direct effect (VOC1 → PIU) is not statistically significant (*P* = 0.316 and 95% CI −0.112 to 0.035), whereas the indirect mediation (VOC1 → psychosocial malaise → PIU) effect, with an estimated coefficient equal to 0.134, is statistically significant (*P* = 0.000 and 95% CI 0.101–0.171), showing a situation of indirect-only mediation.

**Table 11:** Bootstrap results for the direct and indirect mediation effects of VOC1

	Original sample	Sample mean	s.d.	*t*-statistic	*P*	95% CI
Psychosocial malaise → PIU	0.351	0.354	0.036	9.646	0.000	(0.269 to 0.417)
VOC1 → psychosocial malaise	0.365	0.368	0.034	10.782	0.000	(0.292 to 0.425)
VOC1 → PIU	−0.036	−0.037	0.036	1.007	0.316	(−0.112 to 0.035)
VOC1 → psychosocial malaise → PIU	0.134	0.130	0.017	7.490	0.000	(0.101 to 0.171)

PIU, problematic internet use; VOC1, first factorial dimension of the View of Context questionnaire (i.e. absolute unreliability).

**Fig. 3:**
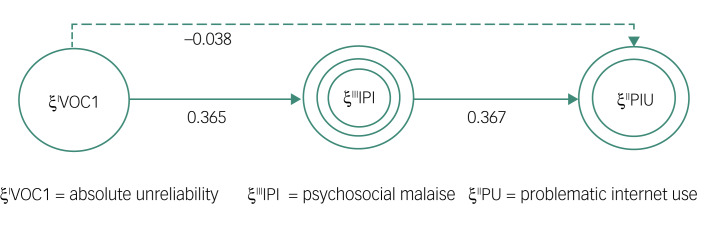
Relationships between components of the model. The dashed line indicates a non-significant effect.

This finding suggests that the mediator (psychosocial malaise) fully complies with the expectation. Fig. 3 shows a graphical representation of the relationship between components of the model.

Thus, psychosocial malaise fully mediates the VOC1 → PIU relationship, with a statistically significant mediation coefficient of 0.134, so our findings empirically support the mediating role of psychosocial malaise in the relationship between absolute unreliability and PIU.

Table 12 shows the bootstrap results for the direct and indirect mediation effects of VOC2. The direct effect (VOC2 → PIU) is not statistically significant (*P* = 0.315 and 95% CI −0.137 to 0.040), and the indirect effect (VOC2 → psychosocial malaise → PIU), with an estimated coefficient equal to −0.027, is not statistically significant (*P* = 0.114 and 95% CI −0.059 to 0.009).

**Table 12:** Bootstrap results for the direct and indirect mediation effects of VOC2

	Original sample	Sample mean	s.d.	*t*-statistic	*P*	95% CI
Psychosocial malaise → PIU	0.343	0.346	0.035	9.760	0.000	(0.271 to 0.407)
VOC2 → psychosocial malaise	−0.075	−0.075	0.047	1.595	0.111	(−0.166 to 0.021)
VOC2 → PIU	−0.043	−0.044	0.045	0.962	0.315	(−0.137 to 0.040)
VOC2 → psychosocial malaise → PIU	−0.027	−0.026	0.016	1.617	0.114	(−0.059 to 0.009)

PIU, problematic internet use; VOC2, second factorial dimension of the View of Context questionnaire.

Thus, by contrast, psychosocial malaise does not mediate the VOC2 → PIU relationship, with a statistically non-significant mediation coefficient of −0.027, indicating a lack of empirical support for the mediating role of psychosocial malaise in the model and the relationship between moderate evaluations of the social environment and PIU.

## Discussion

The present study aimed to investigate the relationship between the way adolescents interpret their social context (i.e. their LDSs) and PIU, considering this relationship mediated by psychosocial malaise. Our hypothesis was that a view of the context underpinned by homogenising affect-laden interpretations, inconsistent with social tasks, rules and goals, may fuel negative affect, which in turn increases the need to indulge in internet use.

The results support this model, demonstrating the role of the latent dimensions of sense in predicting PIU through the mediation of high levels of psychosocial malaise.

As concerns the first hypothesis – adaptive responses are a function of the variable degree of affective salience of the generalised meanings comprising the LDSs – the findings showed that an LDS sense corresponding to a polarised way of viewing the context is associated with adolescents’ PIU through the mediation of psychosocial malaise in terms of high social anxiety, loneliness and negative emotions.

More specifically, the adolescents’ tendency to express negative and extreme evaluations of the social environment (the ‘absolute unreliable’ polarity of the LDS labelled ‘models of relationship with the social environment’) is associated with higher psychosocial malaise, which, in turn, is associated with higher levels of PIU. Accordingly, adolescents’ psychosocial malaise can be understood as reflecting the way of perceiving the sociocultural environment as completely lacking in people and institutions to count on. Within this scenario, there is no hope in the future and the only possibility to get by seems to be by breaking rules, living without scruples and forming alliances with the strongest. As responses on the Likert scale indicate, connotations and evaluations of their environment are rigid, polarised and extremely negative: the variability of objects or events of the experience is not recognised; instead, everything is viewed in a homogenising way. This means that evaluations are not based on an analytic representation of the object, but projected in a generalised category (i.e. the category of ‘good things’ versus the category of ‘bad things’), expressed also with the ‘affect-as-information’ concept.^44^ Accordingly, affect is embodied information about the value and importance of a particular object/stimulus, and such affective valence plays a role in how people judge and then behave. Consistent with our findings, previous studies highlighted how participants who had reactive, extreme and negative attitudes towards the micro and macro social environment were at higher risk of developing pathological gambling.^17,18^ According to our perspective, this reactive tendency of adolescents to express very negative evaluations, homogenising objects and situations, can be interpreted as a sign of an intense affective activation (the same thing happens to a very upset person who sees everything around as a stimulus for their anger). This intense affective activation compromises the regulation of one's thoughts, desires and beliefs and makes it more difficult to act consistently with social constraints and requirements.^19^

Other studies, framed within the theory of mind,^45^ highlight the role of mentalisation or reflective function in behavioural addictions: considering mentalisation as the capacity to recognise one's own and others’ feelings, thoughts, desires and intentions, adolescents with problematic gaming, gambling and social network use^46,47^ seem to have deficits in their reflective function. Such deficits, namely assumptions with little or no correspondence to observable evidence, besides not taking into account the mind's complexity, are strictly associated with emotion regulation problems^46^ and may result in difficulties in regulating one's behaviour. Similarly, other cognitive studies on the link between emotions, cognitions and behaviour^48^ highlighted that the more a person shows poorly differentiated emotion, the more they are unable to respond flexibly to events, ultimately showing maladaptation and malaise.

The role of VOC1 in promoting psychosocial malaise and, in turn, PIU is also consistent with the second hypothesis – adaptive responses are a function of the coherence between the latent dimensions of sense and the role demands made on the participants by the social environment. Indeed, exclusively negative terms aggregated by the ‘absolute unreliability’ polarity are used to describe the relationship with the social environment. The feeling of an adolescent positioned here and expressing higher social malaise and a higher level of PIU is that nobody makes an effort to improve the present and the future of the country, not the ordinary people, not the politicians, not other institutions, so that ‘you can only live day by day’. Conversely, the feeling of congruence between the individual's demands and the environment's responses (as in the ‘moderate reliability’ polarity) works as a protective factor for adolescents, preventing them from suffering and from using the internet as a maladaptive compensation strategy. The findings are consistent with previous studies conducted among young adults and adults in which gambling, drinking and PIU^39,40^ were associated with a negative and anomic view of the social environment, suggesting that social disruption can encourage problematic, maladaptive behaviours as a sort of reaction to this situation. Other scholars have found problematic behaviours in association with antisociality and hostility.^49^

These findings are consistent with Dressler's^23^ cultural consensus theory – arguing that negative health outcomes can be the result of the lack of approximation between an individual's behaviours and beliefs and the cultural models that surround them – as well as in sociological and anthropological studies.^50^ These suggest that problematic behaviour among adolescents is not the result of an existential crisis, or the mere outcome of developmental characteristics which make them ‘naturally’ vulnerable. Rather, it should be attributed to the present-day condition characterised by rapid change, instability and an uncertain future. This leads to a state of anomie, which is characterised precisely by ‘the general idea that the absence of clear rules of behaviour and ambiguity in rules and goals create a state where the individual faces uncertain, conflicting expectations and ambiguous norms and values’.^51^ Growing feelings of uncertainty and ambivalence seem to be the main characteristics of our time, often described as ‘liquid modernity’:^52^ social structures – such as education, health, social security and family – appear fluid, unable to hold their shape. The related lack of solid bonds with no clear line between public and private lives also influences media consumption among youths,^53^ in terms of constant consumption with no clear beginning or end.

From the perspective of semiotic cultural psychology theory, a condition of uncertainty leads sense-makers to adopt affect-laden, homogenised world-views as a way to re-establish stability,^14^ as suggested by the extreme responses of our sample.

The findings also showed a lack of a direct or mediated relationship between the LDS labelled ‘modes of evaluating the social environment’ and PIU. The polarity covers moderate positions in the evaluation of social services (e.g. public offices, health services) and institutions (e.g. the education system, police) and an average proximity to moral values (e.g. the importance of learning and respecting rules). From this perspective, the findings support the idea that maladaptation may not emerge if the adolescent recognises the relevant specificity of the objects/events of the experience and is able to produce differentiated meanings. As for the idealisation polarity, despite the extremism of the response modality, in this case, the social environment is perceived as a comfortable and reliable place – that is, compatible with the individual's demands and needs – and therefore use of the internet as a way to cope with psychosocial malaise does not occur.

### Limitations

Some methodological limitations of the present study have to be considered. First, a convenience sample was used so the results need to be seen in relation to the specific cultural context under analysis, that is southern Italy. In other countries, characterised by different social conditions, other LDSs may emerge. Second, owing to the cross-sectional nature of the study it is not possible to make causal inferences from our data. Third, the study did not take into account the role of potential variables such as perceived social support and sense of community, which may shed further light on how LDSs vary across social segments; the role of emotion dysregulation should be better taken into account because of the recognised part played by intense affective activation; the specific activity conducted online (e.g. social network use, online gaming) should be considered in future research too.

## Data Availability

The data that support the findings of this study are available from the corresponding author on reasonable request.

## Fundings

This research received no specific grant from any funding agency, commercial or not-for-profit sectors.
